# Flagellum tapering and midpiece volume in songbird spermatozoa

**DOI:** 10.1002/jmor.21524

**Published:** 2022-10-28

**Authors:** Emily R. A. Cramer, Gaute Grønstøl, Jan T. Lifjeld

**Affiliations:** ^1^ Natural History Museum University of Oslo Oslo Norway

**Keywords:** mitochondrion, Passerides, postcopulatory sexual selection, scanning electron microscopy, sperm morphology, sperm motility, sperm ultrastructure

## Abstract

In contrast to numerous studies on spermatozoa length, relatively little work focuses on the width of spermatozoa, and particularly the width of the midpiece and flagellum. In flagellated spermatozoa, the flagellum provides forward thrust while energy may be provided via mitochondria in the midpiece and/or through glycolysis along the flagellum itself. Longer flagella may be able to provide greater thrust but may also require stronger structural features and more or larger mitochondria to supply sufficient energy. Here, we use scanning electron microscopy to investigate the ultrastructure of spermatozoa from 55 passerine species in 26 taxonomic families in the Passerides infraorder. Our data confirm the qualitative observation that the flagellum tapers along its length, and we show that longer flagella are wider at the neck. This pattern is similar to mammals, and likely reflects the need for longer cells to be stronger against shearing forces. We further estimate the volume of the mitochondrial helix and show that it correlates well with midpiece length, supporting the use of midpiece length as a proxy for mitochondrial volume, at least in between‐species studies where midpiece length is highly variable. These results provide important context for understanding the evolutionary correlations among different sperm cell components and dimensions.

## Graphical abstract

Across 55 Passerides species, we find that longer sperm flagella are wider at the neck, as previously documented in mammals and reptiles. This result suggests a widespread constraint on the mechanical strength of longer flagella. For interspecific comparisons, sperm midpiece length is an adequate proxy for mitochondrial helix volume, despite the tapering of the helix and flagellum, and despite the fact that the interval between wraps of the mitochondrial helix is longer for longer cells.

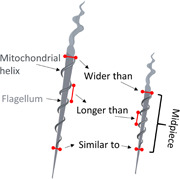

## 1 INTRODUCTION

Spermatozoa of many animals can simplistically be described as consisting of a head, midpiece, and flagellum; variation in the detailed morphology and size of these components results in exceptionally high total diversity of sperm morphology (Kahrl et al., 2021; Pitnick et al., 2009). Substantial work has focused on the relative lengths of these components, showing, for example, that spermatozoa are longer, and length evolves more quickly, in internally fertilizing species (Kahrl et al., 2021); that spermatozoa are longer and less variable among conspecific males where females more often copulate with multiple males (internal fertilizers, e.g., Calhim et al., 2007; Immler et al., 2008; Lifjeld et al., 2010; Tourmente et al., 2011), and that sperm length coevolves with the length of females' sperm storage organs (Briskie et al., 1997; Higginson et al., 2012; Miller & Pitnick, 2002). Sperm length, or the length of individual components, has also been linked to motility in several taxa (e.g., Lüpold et al., 2009; Tourmente et al., 2011; but see Cramer, Garcia‐del‐rey, et al., 2021 and additional references therein), with motility in turn being highly related to fertilization success, both in noncompetitive contexts such as in human couples (Zinaman et al., 2000), as well as in competitive contexts where spermatozoa from more than one male is inseminated (Birkhead et al., 1999; Denk et al., 2005; Donoghue et al., 2003; Gage et al., 2004; Gasparini et al., 2010; Liljedal et al., 2008; Pizzari et al., 2008). In contrast to the large body of research on spermatozoa length, and recent detailed work on sperm head morphology (Hook et al., 2021; Støstad et al., 2018), relatively few studies focus on the width of sperm midpiece and flagellum, although width may impact the mechanical strength of the cell (Baltz et al., 1990; Lindemann & Lesich, 2016) and the volume of the cell devoted to particular physiological processes such as ATP production (Gu et al., 2019). Evaluating the width of the midpiece and flagellum is therefore an important step in understanding how spermatozoa function, and potentially in understanding the evolution of sperm length.

The midpiece generally begins at the tail‐end of the head and encloses or wraps around a portion of the flagellum (Cummins, 2009). It is the portion of the cell housing the mitochondria, and thus is responsible for ATP production via oxidative phosphorylation. Midpiece volume may determine the total amount of ATP produced by the cell and in turn affect swimming performance (Gu et al., 2019). Consistent with higher demands for sperm swimming ability in species with higher sperm competition, midpiece volume is greater in species where females copulate with multiple males (Anderson & Dixson, 2002; Anderson et al., 2005), a pattern also found in some but not all studies using midpiece length as a proxy for midpiece volume (Immler & Birkhead, 2007; Immler et al., 2007; Lüpold et al., 2009; Rowley et al., 2019; Tourmente et al., 2011). The flagellum, in turn, uses the chemical energy from ATP to change shape and thus to push the cell forward by exerting force against its environment (Cummins, 2009; Lindemann & Lesich, 2016). Depending on the species and circumstances, the flagellum may also create ATP via glycolysis (Cummins, 2009; Ford, 2006). The size and physical arrangement of the midpiece and flagellum, therefore, have important consequences for motility, for example, potentially affecting the amount of ATP that can be synthesized (Rowe et al., 2013; Tourmente et al., 2013), the transport needs for moving ATP from its site of synthesis to where it is used (Cardullo & Baltz, 1991; Villar et al., 2021), and in the physical strength of the cell and its ability to translate waves or bends in its structure into forward motion via interactions with the environment (Baltz et al., 1990; Lindemann & Lesich, 2016). While the structure and length of the midpiece and flagellum have been highly studied in mammals and some externally fertilizing invertebrates, relatively less is known in other taxa. In this study, we detail the width and physical arrangement of the midpiece and flagellum of 55 species in 26 taxonomic families of Passerides songbirds.

**Figure 1:**
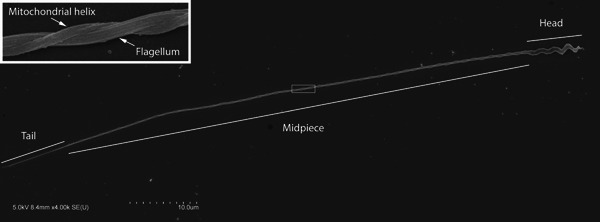
Image of a marsh warbler (*Acrocephalus palustris*) sperm cell at 4000× magnification (several images stitched together; see Section 2 for details; empty spaces filled with gray). The head (including acrosome and nucleus), midpiece, and tail (i.e., exposed flagellum) are marked. The inset shows a zoomed‐in image of the portion of the cell indicated by the box, consisting of just more than 1 gyre (i.e., complete wrap of the mitochondrial helix). Note that the midpiece and tail are regions of the cell, while the flagellum and mitochondrial helix are each structures. The presence of the mitochondrial helix defines the limits of the midpiece. The flagellum, while also present in the midpiece, extends beyond it, in the tail of the cell. Measures of midpiece length more closely trace the center of the midpiece compared to the straight‐line distance shown here (e.g., Cramer et al., 2020).

Within the oscine passerines, and particularly within the infraorder Passerides, the general structure of the midpiece and flagellum is fairly conserved (Figure 1). There are, however, notable exceptions (Birkhead et al., 2006; Lifjeld et al., 2013) and high variability in the length of the cells and the individual components. As in many organisms, the core of the flagellum is the axoneme, a set of 9 microtubule doublets arranged radially around a central pair of single microtubules (a 9 + 2 configuration; Henley et al., 1978; Jamieson, 2007). Dynein arms in these microtubules drive motility. For most of its length, each doublet microtubule has an associated keratinous outer dense fiber (ODF), which tapers along the flagellum and disappears toward the tail (Aire et al., 2017; Henley et al., 1978; Jamieson, 2007; Jamieson et al., 2006). In the midpiece, an elongated mitochondrion (formed by the fusion of many mitochondria during spermatogenesis) with a roughly cylindrical cross‐sectional shape wraps helically around the flagellum. Thus, much of the surface of the flagellum is not covered by the mitochondrial helix, although in some species the mitochondrion is briefly shaped like a ring at the base of the neck, before quickly becoming more of a cylinder spiraling around the flagellum (Jamieson, 2007). The midpiece (i.e., the portion of the cell where the mitochondrial helix wraps around the flagellum) can constitute quite a large proportion of the flagellum (74.2 ± 20.9%, mean ± SD for the 264 passerines described by Omotoriogun et al., 2020). There is positive allometry between the length of the mitochondrial helix and the flagellum length (1.76 for 50 passerine species in 4 taxonomic families, Immler & Birkhead, 2007). However, the cross‐sectional area of the mitochondrial helix is negatively correlated with length in the one species where that has been addressed (zebra finch, *Taeniopygia guttata*, Mendonca et al., 2018), so that the volume seems rather constant for different lengths of the helix in that species. It is therefore unclear how midpiece length relates to mitochondrial volume in general across Passerides songbirds. Many studies have investigated how the length of the midpiece and/or flagellum correlate with swimming speed, but no general pattern has been found in Passerides songbirds (e.g., Cramer et al., 2015; Cramer, Garcia‐del‐rey, et al., 2021; Helfenstein et al., 2010; Immler et al., 2010; Kleven et al., 2009; Lüpold et al., 2009; McDiarmid et al., 2022; Rojas Mora et al., 2018).

Additional structures are also known in the midpiece of Passerides songbirds, although their function is generally not well known. For example, a fibrous helix may wrap around the flagellum either peripherally (forming a keel‐like structure; in the European blackbird *Turdus merula*) or centrally (in Southern anteater‐chat *Myrmecocichla formicivora* and European starling *Sturnus vulgaris*) to the mitochondrial helix (Furieri, 1961; Jamieson, 2007; Jamieson et al., 2006; Vernon & Woolley, 1999). A granular helix occurs in some species (Aire, 2014; Jamieson, 2007; Tripepi & Perrotta, 1991) and can either encase part of the mitochondrial helix just posterior to the head, or it can replace the first portion of the mitochondrial helix (Jamieson, 2007). Whether the proximal centriole is retained also varies (Aire, 2014; Sotelo & Trujillo‐Cenóz, 1958); all species retain the distal centriole (Jamieson, 2007). These structures are difficult to identify with certainty using scanning electron microscopy (SEM); the focus of this paper is primarily on the mitochondrial helix and flagellum.

To understand the function of various structures, and to generate predictions, we draw on the literature on mammal spermatozoa. Flagellum structure is generally similar in mammals, though the arrangement of the midpiece is quite different. Like birds, mammals generally have a 9 + 2 axoneme structure with associated ODFs (Fawcett, 1970). The ODFs are hypothesized to add structural strength against shearing, providing support during ejaculation and swimming (Baltz et al., 1990; Lindemann & Lesich, 2016; Phillips, 1972). As in Passerides, ODFs taper along the flagellum (Fawcett, 1970; Gu et al., 2019), and they are thicker at the head end in mammal species with longer spermatozoa (Gu et al., 2019; Zhao et al., 2018). Relative to the flagellum, the midpiece is generally shorter in mammals than in Passerides (proportion of flagellum encompassed by midpiece in 194 eutherian mammals, mean ± SD, 22.1 ± 6.4%, maximum 49.4%, Tourmente et al., 2011). The arrangement of the midpiece relative to the flagellum is also quite different in mammals, where the midpiece consists of individual, closely aligned mitochondria that together create a sheath encompassing the full circumference of the flagellum. Despite these differences, allometry is roughly similar to birds, with a scaling parameter of approximately 1.5 (though note that this analysis was not corrected for phylogenetic relatedness, Cardullo & Baltz, 1991). Similarly to zebra finches, there may be a negative correlation between midpiece length and mitochondrial cross‐sectional area in the midpiece (one study found that midpiece volume increases more slowly with flagellum length than midpiece length does, Cardullo & Baltz, 1991; while another study found midpiece volume to be unrelated to flagellum length, Gage, 1998). The cross‐sectional area of the mammalian mitochondria also tapers along the midpiece length (Gu et al., 2019). Mammalian spermatozoa have an additional fibrous sheath that wraps a portion of the flagellum, caudal to the midpiece, but this is lacking in Passerides songbirds.

In this paper, we examine the allometry of midpiece and flagellum lengths in Passerides. We describe the flagellum diameter, how it tapers from head to tail, and whether it correlates with flagellum length, as may be expected from work in mammals. We further characterize the structure of the mitochondrial helix and its physical relationship to the flagellum. Specifically, we estimate two diameters for this helix, quantify how it tapers and how it wraps around the flagellum, and estimate mitochondrial volume. Understanding whether midpiece length can be used as a proxy for mitochondrial volume is important to understand previous papers' results about ATP generating capacity (Bennison et al., 2016; Immler & Birkhead, 2007; Rowe et al., 2013; Yang et al., 2020). Finally, based on the result of Støstad et al. (2018) that sperm head shape affects swimming speed and correlates with sperm total length, we ask whether the diameter of the sperm nucleus relates to the diameter of the flagellum.

## 2 MATERIAL AND METHODS

Sperm samples were taken from the Natural History Museum of the University of Oslo Avian Sperm Collection (Lifjeld, 2019) and had all been stored in 5% buffered formaldehyde (see full species list in Supporting Information: Table S1; naming of species follows the IOC World Bird list 11.2). Individuals were chosen based on the availability of samples with a high density of spermatozoa with little debris. Species were chosen for analysis somewhat haphazardly, largely using specimens that had already been prepared for SEM for an earlier study (Støstad et al., 2018; as a cost‐ and labor‐saving way to build the data set; *n* = 40 species). We chose new species (*n* = 15 species) to add to the data set with the goal of representing a range of sperm lengths within and between taxonomic groups. Cells were prepared for SEM either in 2016–2017 (species included and/or evaluated for inclusion in Støstad et al., 2018) or in 2022 (Supporting Information: Table S1). For each sample, 50 µl was placed on a poly‐lysine‐coated cover slip. Samples prepared in 2022 were first washed in PHEM buffer (Schliwa & Van Blerkom, 1981). All samples were then dehydrated using a series of increasing ethanol concentrations. Critical point drying was performed with a BAL‐TEC CPD 030 Critical Point Dryer. After mounting the cover slips on stubs using carbon tape, they were sputter‐coated with platinum (5–13 nm; Cressington 308R) and imaged with Hitachi S‐4800 field emission SEM operated at 5.0–7.0 kV.

To hold magnification and image resolution across species, overlapping (4000× magnification) images were captured along the whole cell length and stitched together (Preibisch et al., 2009); see the example in Figure 1. Images were taken in 2021 (from samples prepared in 2016–2017) or in 2022 (from freshly‐prepared samples). We found no evidence of changes in the length of the midpiece or the tail due to the storage of prepared samples for 5 years (see Supporting Information for details), nor did we notice elevated damage in cells from the older preparations. We chose cells that showed no damage to the mitochondrial helix, with minimal dust or other contamination to obscure the midpiece. Because such cells could be difficult to find, we included cells with damaged acrosomes and nuclei. Because our primary measurements were on the midpiece, we also included some cells where it appeared that the tip of the flagellum, at the distal end of the cell, was blunt rather than tapering to a very small tip, as a relatively large proportion of cells had this appearance. The end of the mitochondrial helix was typically either rounded or smoothly tapering, allowing us to exclude cells where the mitochondrial helix was broken prematurely. Comparison of measurements from these cells with light microscopy measurements (see below and Supporting Information: Tables S1 and S2) suggest that these cells are fairly representative of the individual's sperm length. In addition, we investigated two other sets of SEM images, which either had relatively low image quality or lacked positional information, but that included some replication within males and multiple males per species (detailed in Supporting Information). As these alternate datasets with higher replication showed overall similar results, we suggest that the overall patterns we observe are robust, even if some measured cells by chance are not ideal representatives for that male/species.

### 2.1 Flagellum and midpiece measurements

**Figure 2:**
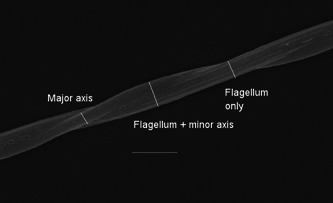
Reed bunting (*Emberiza schoeniclus*) flagellum and mitochondrial helix, showing approximately one full gyre and indicating the types of measurements taken. Scale bar is 1 μm. This section of the mitochondrial helix appears ellipsoid in cross‐section.

We took three types of measurements with the straight‐line tool in ImageJ (Schneider et al., 2012; Figure 2). Pilot testing indicated that it was not feasible to directly measure the minor axis of the mitochondrial helix (sensu Mendonca et al., 2018). We therefore instead measured the width of the flagellum (i.e., minimum width per half‐gyre) and the width of the flagellum and mitochondrial helix (i.e., maximum width per half‐gyre), and determined the minor axis diameter by subtraction. These measures were both taken perpendicular to the long axis of the flagellum (Figure 2). The width of the major axis of the mitochondrial helix was taken perpendicular to the long axis of the mitochondrial helix. The latter measurement was more sensitive to image quality and thus it was not possible to measure for all of the cells. We took each of the measurements at each opportunity along the cell (i.e., all clear flagellum and flagellum + minor axis widths were measured, yielding more measurements per cell for longer cells), beginning at the head and working toward the tail. Accurate measures of flagellum width could not be made at the neck for cells with additional structures or a ring‐shaped mitochondrial helix there (investigated in Supporting Information). We therefore began measuring posterior to this region in cells where it was obscured by other structures.

For the relatively few locations where debris on the stub or gross abnormalities or breakages in the cell prevented measurements of the flagellum or flagellum + minor axis, we marked the longitudinal position of the measurement point, if possible. In cases where debris fully obscured a section of the cell, we measured the length of that section with the segmented line tool, so that the number of missed gyres could be estimated analytically.

We extracted positional information from the measurement data set, allowing us to estimate the length of each gyre as the distance between the center points of corresponding measurements from consecutive gyres. Further, we measured the length of the midpiece and the tail (exposed flagellum) using the segmented line tool. These measurements are equivalent to our lab's measurements on light microscopy images (detailed in the next section). The flagellum length was calculated as the sum of the midpiece and tail. Finally, we measured the diameter of the nucleus. For species where the nucleus appeared to taper, the measurement was taken immediately anterior to the apparent start of the tapering. One person took all SEM measurements.

In total, we obtained 2031 pairs of flagellum and flagellum + minor axis measurements from 55 males, each of a different species. We further measured 952 major axes from 53 males (note that ideally there would be half as many major axis measurements as pairs of other measures).

### 2.2 Light microscopy

To explore allometry of the flagellum and midpiece, we used data from additional individuals (where available) of each species, with measurements taken using light microscope images (Supporting Information: Table S1). About 15 µl of formalin‐fixed sperm sample was streaked onto a microscope slide, allowed to dry overnight, rinsed with distilled water, and then imaged at 320× magnification (Leica DM6000B and DC420). The length of the head, midpiece, and tail was measured using Leica Application Suite v. 4.1.0 (Leica Microsystems), generally for 10 cells per male. No stains were used, as transitions between the head, midpiece, and tail are visible without staining, given sufficient practice. Under light microscopy, the midpiece often appears somewhat striated due to the wrapping of the mitochondrial helix (though variation in the thickness, i.e., the flagellum vs. flagellum + minor axis measures, are not clearly visible). The midpiece is also generally noticeably thicker than the tail, with a relatively abrupt transition in thickness where the midpiece ends. Images of the same field of view, taken at slightly different focal planes, can further help to distinguish the transitions between segments.

Measurements of the midpiece and tail length were taken by several observers. While measurement repeatability was not directly assessed for the samples in this study, previous work in our group shows high measurement repeatability: 94%–98% for midpiece and 80%–95% for tail (Cramer et al., 2020; Cramer, Grønstøl, et al., 2021; Laskemoen et al., 2010). We assessed how mean light microscopy measures from the same individual males correlated with SEM‐based length measurements (Supporting Information: Table S2).

### 2.3 SEM measurement accuracy and bias

To evaluate repeatability of SEM measurements, we marked a haphazardly chosen subset of gyres to be remeasured for major axis, flagellum, and flagellum + minor axis. We marked and remeasured (4 measurements total, all on different days) 3 different gyres per cell for 4 cells. We estimated repeatability using rptR assuming a Gaussian distribution (Stoffel et al., 2017), including measurement type (major axis, flagellum, or flagellum + minor axis) as a fixed effect. Repeatability was 0.998 (SE 0.001, confidence interval [CI] = 0.996–0.999, *p* < .001 The difference between the largest and smallest values for repeated measurements of the same point was 3.8 ± 2.6% (mean ± SD of the absolute value of the difference).

It was not possible to blind measurements with respect to position along the cell or to midpiece length. However, to minimize potential bias, the window containing measurement results was positioned to make measured lengths not visible during the measuring. Most images (40 cells) were named such that species information was not apparent. For 15 cells prepared for SEM in 2022, species names were visible during the measurement of diameters. We contend that this was unproblematic, as there were no strong expectations linked to species identity.

### 2.4 Estimating mitochondrial helix volume

Flagellum and mitochondrial helix widths changed depending on their longitudinal position in the cell (see Section 3). To account for this change, we estimated the mitochondrial helix volume for each gyre separately and then summed across all gyres in the cell. Following the logic of Mendonca et al. (2018), we approximated the cross‐section of the mitochondrial helix as an ellipse, so that cross‐sectional area can be calculated as the product of the major axis radius, minor axis radius, and π. We had one major axis measurement per gyre, since this could only be measured when the mitochondrial helix was above the flagellum, but generally two minor axis measures per gyre; we used the mean of the two minor axis measures. We then multiplied the cross‐sectional area by the length of the mitochondrial helix within the gyre. This length was calculated as the square root of the sum of gyre length squared and flagellum circumference squared (Birkhead et al., 2005). Flagellum circumference was calculated as π × diameter, where the diameter was the average of the two flagellum width measurements for the gyre. In cases where one type of measurement was missing due to debris, we used the average value for the whole cell to replace the missing value. For cells where complete gyres were not measurable, we estimated the number of missing gyres based on the length of the unmeasurable section, and we assigned the mean per‐gyre volume to these missing gyres.

### 2.5 Statistical analysis

#### 2.5.1 Phylogeny

We downloaded 1000 trees with our 55 species of interest from birdtree.org, specifying the Hackett backbone. The consensus tree with branch lengths was found with the least squares method (Revell, 2012). We used a Bayesian approach in the package MCMCglmm (Hadfield, 2010) as it conveniently allows for mixed effect models in a phylogenetic framework. For consistency, we also used MCMCglmm for simpler models using only single values per species (results were similar using a frequentist approach in package ape (Paradis et al., 2004), not shown).

#### 2.5.2 Length allometry

We explored the allometric relationship between midpiece and flagellum length using measurements from light microscopy, since these measurements represent the greater number of individuals and cells. We first calculated the mean across the multiple cells per male and then among multiple males per species. For consistency with previous studies of allometry, we log‐transformed both measurements (with natural logarithm), and used midpiece length as the response variable with flagellum length as the predictor.

#### 2.5.3 Change in measures along the flagellum, and relationships with flagellum length

To investigate how measures changed across the length of the flagellum, and how they relate to total flagellum length, we ran separate models for each of four response variables: flagellum width, mitochondrial helix minor axis width, mitochondrial helix major axis width, and the interval between successive gyres. The response variable was log‐transformed only if doing so improved the normality of residuals from the models (marked as “a” in Table 1; natural logarithm used). Each measurement along the cell was included as an observation. Predictors were flagellum length for that individual cell (to estimate the relationship between flagellum length and the width measures) and longitudinal position (to assess change across the flagellum). The longitudinal position was expressed by converting the ordinal measurement number to a proportion. The measured gyre closest to the head, therefore, had a value of 0 and the closest to the tail had a value of 1 for all cells; this was done to avoid having an uneven distribution of longitudinal positions due to long cells having many measured gyres. Flagellum length was mean‐centered (Schielzeth, 2010) and divided by 100 to produce numerically convenient parameter estimates (i.e., to avoid many leading 0's in parameter estimates). An interaction term between longitudinal position and flagellum length was initially included but removed if it was nonsignificant. Species (which is redundant with cell identity) was included as a random effect. To evaluate whether the relationship between flagellum diameter and length was driven by differences close to the head or close to the tail, we reran the same model structure but using only the 25% of observations closest to the head or tail (in separate models).

To further understand the tapering of the flagellum, we evaluated whether the diameter of the flagellum just distal to the end of the midpiece (i.e., at the head‐end of the tail) related to the length of the tail. Our logic here was that a wide flagellum at the start of the tail could occur if the mitochondrial helix stops relatively early on the flagellum, such that the flagellum continues to taper over the length of the tail. We a priori used mean tail values from light microscopy measures of the same individuals, as some cells that were used for SEM measuring had slightly truncated tails (we assumed that this contributes minimal noise to flagellum length, but may contribute more substantially to tail length; see Supporting Information: Table S2).

#### 2.5.4 Mitochondrial helix volume

To evaluate how mitochondrial helix volume relates to midpiece length measurements, we used mitochondrial helix volume as the response variable and midpiece length (from the segmented line measurement on the SEM image of the same cell) as the predictor. We a priori chose to analyze only the SEM midpiece length, but given the high correlation between midpiece length measures from the SEM data set and from light microscopy (Supporting Information: Table S2), we expect that relationships among variables would be essentially identical with midpiece length from light microscopy. Because our goal is to assess whether light microscopy measures of midpiece length are an adequate proxy for midpiece volume, we include the full length of the midpiece (i.e., including any gyres at the neck end where widths were not measured), since this section is indistinguishable from the rest of the midpiece under light microscopy.

#### 2.5.5 Nucleus diameter and flagellum

We examined whether the diameter of the nucleus correlated with the first three measurements of the flagellum diameter. Here, we arbitrarily and a priori chose to use the mean of the first three measurements as the response variable. Using the first three measurements attempted simultaneously to reduce the impact of potential measurement error (by using more values) while only including values close to the head to avoid tapering effects.

### 2.6 Data accessibility

The data and code that support the findings of this manuscript, as well as the stitched images, are available at Data Dryad with DOI 10.5061/dryad.n5tb2rbzt. All calculations and analyses were conducted in R 4.1.1 (R Development Core Team, 2012); in addition to already‐named packages, we used tidyverse (Wickham et al., 2019) and RcppRoll (Ushey, 2018). Except where otherwise noted, we report mean and 95% CIs calculated from the Bayesian posterior distributions for parameter estimates.

## 3 RESULTS

### 3.1 Length allometry

The allometric relationship between midpiece length and flagellum length (using mean light microscope measurements across additional individuals) was described by the equation log(midpiece length) = −1.99 + 1.38 × log(flagellum length), including all 55 species (CI for intercept [−2.41, −1.63], *p* < .003; for slope [1.30, 1.47], *p* < .003). Residuals were substantially improved by excluding the species with the shortest midpiece (white‐throated dipper, *Cinclus cinclus*), though positive allometry was still evident without this species (log(midpiece length) = −1.55 + 1.29 × log(flagellum length); CI for intercept [−1.84, −1.28], *p* < .003; for slope [1.22, 1.34], *p* < .003). Positive allometry was also evident using a phylogenetic reduced major axis regression (in phytools, Revell, 2012; data not shown).

### 3.2 Change in measures along the flagellum, and relationships with flagellum length

**Figure 3:**
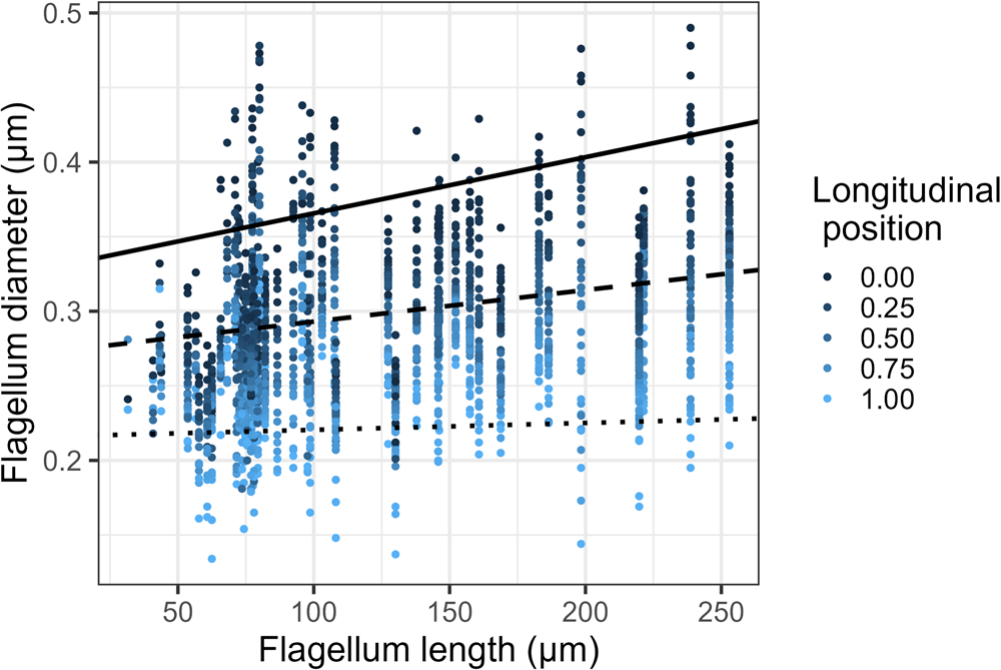
Flagellum diameter change over longitudinal position (color gradient, 0 = neck, 1 = tail), and with total flagellum length. The lines are estimates from the statistical analysis controlling for phylogeny, where longitudinal position is 0 (solid; neck), 0.5 (dashed), or 1 (dotted; tail). Each cell is represented by multiple points (one point per measurement location).

Flagellum diameter decreased along the length of the flagellum and was on average larger for longer flagella (Table 1, Figure 3; Supporting Information: Table S3, S4, and Figure S1). To better understand how diameter related to flagellum length, we examined whether diameter for the 25% of measurements closest to the neck (proximal) and, separately, the 25% of measurements closest to the tail (distal) for each cell correlated with flagellum length (with no other covariates, only control for phylogeny, as for the main model). Diameters near the neck correlated positively with flagellum length, indicating that longer flagella begin with a wider diameter. Tail‐end diameters within the midpiece did not correlate with flagellum length, indicating that tail‐end flagellum diameter is similar across flagella lengths. This pattern is also evident from the interaction term in the model with the full data set (Figure 3).

**Table 1:** Statistical results relating flagellum diameter, the major or minor axis diameters of the mitochondrial helix, and the gyre interval of the mitochondrial helix to longitudinal position along the cell, and flagellum length

Response (data set)	Predictor	Estimate [CI], statistics	*λ* (mean [CI])
Flagellum diameter (all measures)	(Intercept)	0.33 [0.28, 0.37], df = 394, *p* = .003	0.85 [0.79–0.90]
	Longitudinal position	−0.11 [−0.12, −0.11], df = 394, *p* = .003	
	Flagellum length	0.04 [0.02, 0.06], df = 394, *p* = .003	
	Flagellum length × longitudinal position	−0.03 [−0.04, −0.03], df = 535, *p* = .003	
Flagellum diameter (proximal 25%)	(Intercept)	0.31 [0.27, 0.37], df = 394, *p* = .003	0.88 [0.84–0.93]
	Flagellum length	0.04 [0.02, 0.07], df = 394, *p* = .01	
Flagellum diameter (distal 25%)	(Intercept)	0.23 [0.20, 0.27], df = 394, *p* = .003	0.79 [0.71–0.85]
	Flagellum length	−0.00 [−0.02, 0.01], df = 394, *p* = .79	
Minor axis of mitochondrial helix^a^	(Intercept)	−1.87 [−2.16, −1.56], df = 412, *p* = .003	0.66 [0.57–0.75]
	Longitudinal position	−0.51 [−0.55, −0.47], df = 394, *p* = .003	
	Flagellum length	−0.04 [−0.18, 0.10], df = 394, *p* = .53	
Major axis of mitochondrial helix^a^	(Intercept)	−1.44 [−1.61, −1.25], df = 394, *p* = .003	0.57 [0.46–0.71]
	Longitudinal position	−0.25 [−0.28, −0.21], df = 394, *p* = .003	
	Flagellum length	−0.07 [−0.16, 0.02], df = 394, *p* = .18	
Gyre interval^a^	(Intercept)	1.43 [1.23, 1.61], df = 394, *p* = .003	1.16 [1.09–1.25]
	Flagellum length	0.23 [0.14, 0.33], df = 394, *p* = .003	
	Longitudinal position	−0.08 [−0.09, −0.07], df = 394, *p* = .003	
	Flagellum length × longitudinal position	0.05 [0.03, 0.06], df = 406, *p* = .003	

*Note*: Estimates are means (95% CI) from the posterior distribution of the Bayesian model. Flagellum length was centered and scaled by dividing by 100. Longitudinal position was between 0 and 1 for all cells. Here, df indicates effective sample size after the Markov chain process in MCMCglmm, controlling for shared phylogenetic history.

^a^ The response variable was log‐transformed to improve normality of residuals.

The diameter of the flagellum just after the end of the midpiece was on average 0.19 ± 0.04 µm (mean ± SD; range 0.12–0.28 µm) for the 55 species imaged here (Supporting Information: Table S1). For comparison, the mean diameter after the midpiece in the Eurasian and Azores bullfinches (*Pyrrhula pyrrhula* and *Pyrrhula murina*), which appear to lack ODFs, are 0.17 ± 0.01 and 0.16 ± 0.01 µm, respectively (mean ± SD; measured from 9 to 10 images from one male of each species, using images described in Lifjeld et al., 2013). (Note that one Eurasian bullfinch sperm cell with a flagellum diameter of 0.3 µm was excluded; we assume that this was a double flagellum, as illustrated in Fawcett et al., 1971; and Swan & Christidis, 1987 in other passerines). In our 55 species, the diameter of the flagellum at the start of the tail was positively related to tail length (estimated effect [CI]: 0.004 [0.002, 0.005], *p* < .003) and not related to midpiece length (6 × 10^−5^ [−9 × 10^−5^, 2 × 10^−4^], *p* = .38; intercept 0.12 [0.09, 0.16], *p* < .003; with both predictors included in one model, variance inflation factor <2 in a pilot with phylogenetic generalized least squares approach). Overall, then, it appears as though flagellum width may be similar across species at the tail‐tip end, but increases to a greater diameter on the neck‐end for longer flagella. We did not measure diameters along the tail, because our data set included some imperfect tail‐tips, so we cannot evaluate this model rigorously. The diameter of the flagellum at the end of the midpiece depends on where on the flagellum the midpiece ends, which varies among species.

For both the major axis and the minor axis of the mitochondrial helix, diameter decreased with longitudinal position along the flagellum, and this decrease was not related to flagellum length (Table 1; Supporting Information: Table S4), although in a supplementary data set with lower image resolution and higher measurement error, the minor axis of the mitochondrial helix was smaller for longer cells (Supporting Information: Table S3).

**Figure 4:**
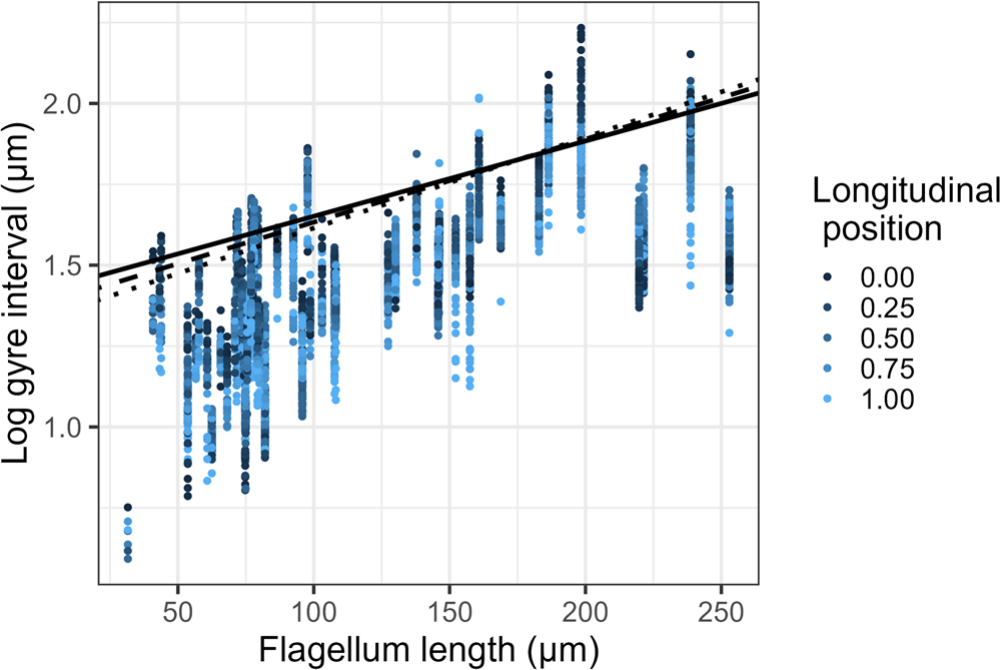
Change in the distance between successive wraps of the mitochondrial helix (gyre interval, transformed with natural logarithm) over the length of the flagellum (longitudinal position, shown with color gradient, 0 = neck, 1 = tail), and with total flagellum length. The lines are estimates from the statistical analysis controlling for phylogeny, where longitudinal position is 0 (solid; neck), 0.5 (dashed), or 1 (dotted; tail). Each cell is represented by multiple points (one point per measurement location).

The number of gyres per cell varied from about 2.5 in the white‐throated dipper to over 40 in the chaffinch (*Fringilla coelebs*), American redstart (*Setophaga ruticilla*), and indigo bunting (*Passerina cyanea*; Supporting Information: Table S1). The interval between gyres ranged from 1.95 µm (white‐throated dipper) to 6.69 µm (blue‐gray tanager, *Thraupis episcopus*; Supporting Information: Table S1). The interval between gyres on average was longer in species with longer flagella, implying fewer wraps of the midpieces per length of flagellum (Table 1, Figure 4; Supporting Information: Table S3). Gyre interval also was significantly affected by longitudinal position along the flagellum, and by an interaction between longitudinal position and flagellum length, although these appeared to explain little variation (Table 1, Figure 4; Supporting Information: Table S3).

### 3.3 Mitochondrial helix volume

**Figure 5:**
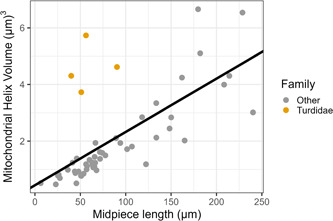
Relationship between midpiece length (i.e., the length of the portion of the cell where the mitochondrial helix wraps around the flagellum) and the estimated volume of the mitochondrial helix. Species in the family Turdidae are in yellow, as they are known to have an additional structure, which our measurements did not distinguish; their mitochondrial volume is therefore not accurately estimated using our protocol. The line shows fitted values from the phylogenetically controlled analysis, including both Turdidae and other species.

**Figure 6:**
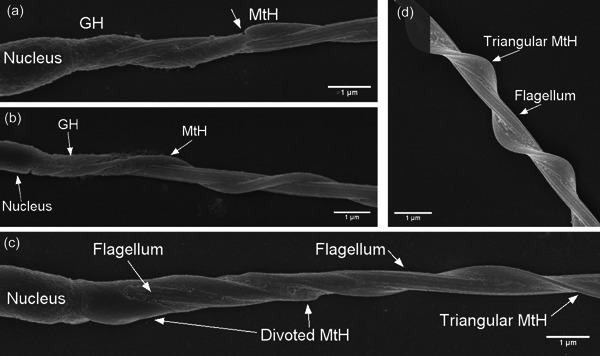
Example images showing putative granular helix (GH) as well as variation in the appearance of the mitochondrial helix (MtH), consistent with the presence of other helical structures. (a) Chaffinch *Fringilla coelebs*, with the GH appearing to replace the MtH, showing a sharp break point at the arrow. (b) Willow warbler *Phylloscopus trochilus*, with the GH encompassing the MtH for a brief portion of the neck; the MtH has a typical elliptical appearance. (c) Bluethroat *Luscinia svecica*, with the MtH appearing first divoted and then triangular. D: American robin *Turdus migratorius*, with a triangular MtH. See more details in the Supporting Information.

Estimated mitochondrial volume correlated strongly and significantly with midpiece length across species (estimate [CI] for midpiece length, 0.02 [0.013, 0.024], *p* < .003; intercept, 0.44 [−0.18, 0.99], *p* = .14, Figure 5). These estimates assume that the helical structure around the flagellum is entirely composed of mitochondria; as described in the introduction, other structures may also occur. Indeed, we observed structures consistent with descriptions of the granular helix (which we avoided including in calculating midpiece volume) and of fibrous helices either peripheral or central to the mitochondrial helix (examples in Figure 6; detailed in the Supporting Information: Table S1; these structures were not possible to separate from the mitochondrial helix with our measurement approach). All species in Turdidae likely have a fibrous helix (Supporting Information: Table S1), since several *Turdus* species are known to have it, and Turdidae mitochondrial helices appeared generally similar. Turdidae species did not drive the positive relationship between midpiece length and volume relationship, as the relationship between midpiece length and volume was similar (though the intercept was lower) after excluding these species (estimate [CI] for midpiece length, 0.02 [0.018, 0.025], *p* < .003; intercept, −0.06 [−0.43, 0.35], *p* = .78).

### 3.4 Nucleus diameter and flagellum

**Figure 7:**
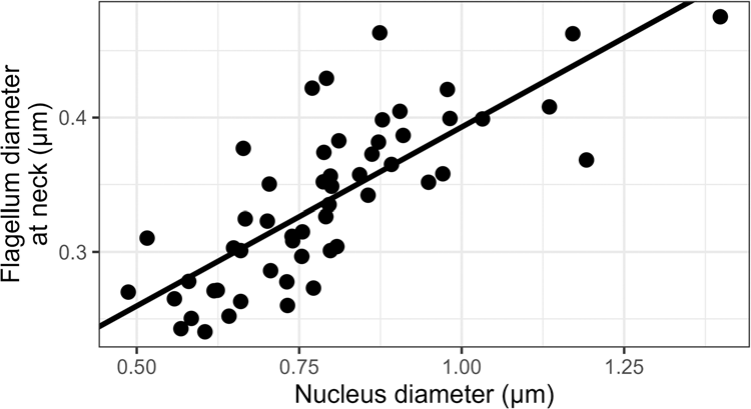
Relationship between the diameter of the nucleus just anterior of the flagellum, and of the flagellum just posterior of the head (i.e., the average of the first three measured diameters of the flagellum). Each point represents one cell (one cell measured per species). Fitted line from the phylogenetically controlled model is shown.

Flagellum diameter at the neck region (first 3 measured diameters) correlated with head diameter, and was smaller than head diameter (Figure 7; model intercept and slope were both significantly positive; 0.13 [0.08, 0.17], *p* < .003 and 0.27 [0.21, 0.33], *p* < .003, respectively).

## 4 DISCUSSION

Despite the differences between songbirds and mammals in ejaculation and sperm cell swimming mechanics, patterns of flagellar structure observed in mammals also hold in Passerides songbirds. That is, longer cells have thicker flagella, particularly toward the cell's head, and the flagellum diameter narrows toward the tail. Though we have not measured which aspects of flagellum structure drive flagellum width, we suspect that it is largely driven by the size of the ODFs, since these structures appear relatively large compared to the axoneme in passerines, as well as appearing to be reduced in area in serial sections of the same cell (based on transmission electron micrograph [TEM] images: fig. 6 in Aire et al., 2017; fig. 1 in Mendonca et al., 2018; fig. 17 in Vernon & Woolley, 1999). Tourmente et al. (2009) find that longer cells have larger ODFs in snakes (though with low sample size, and they measured the area of only two ODF, which are enlarged relative to the other ODF in snakes). If we are correct that our results are driven by ODF diameter, our results may highlight the general importance of the structural support provided by ODFs for longer flagella (Baltz et al., 1990) even under diverse swimming styles. Interestingly, ODFs appear to be absent in the spermatozoa of bullfinches (see fig. 3 in Lifjeld et al., 2013). The similarity in flagellum diameter between bullfinches and the tail‐end measures of the species in this paper suggests that in most of the species studied here, the midpiece ends at approximately the point where the ODFs have ended within the flagellum. However, for some species, the flagellum diameter at the start of the tail was substantially higher, and in such species, there was a greater length of tail over which tapering presumably continues. It therefore appears as if the midpiece stopped at a relatively proximal point on what may be a similar underlying flagellar structure in such species. The evolutionary connection between flagellum length and diameter therefore appears to be stronger than the connection between flagellum structure and the mitochondrial helix (with the latter being the defining characteristic of the midpiece).

Many studies use midpiece length as a proxy for midpiece volume, which in turn is assumed to be a proxy for ATP‐producing capacity. Having an accurate proxy is important: one study in mammals found associations between promiscuity level and midpiece volume, but not between promiscuity level and midpiece length (Anderson et al., 2005). Estimated mitochondrial volume was a strong predictor for ATP production in the cell in mammals (Gu et al., 2019). Our results provide evidence that midpiece length may be a sufficient proxy for mitochondrial volume for interspecific studies on Passerides songbirds, and indeed midpiece length positively correlates with ATP content across Passerides species (Rowe et al., 2013). That is, midpiece volume is tightly correlated with midpiece length across Passerides species, likely due to the high variation in midpiece length among taxa. For studies working at an intraspecific level, or with a set of species with similar midpiece lengths, investigating mitochondrial volume rather than midpiece length may still be warranted; indeed, two intraspecific studies did not find correlations between midpiece length and ATP content (Bennison et al., 2016; Yang et al., 2020). In addition, we have not accounted for two substantial sources of variation in mitochondrial volume: cross‐sectional shape of the mitochondrial helix and the presence of other structures within the midpiece. TEMs show variation from approximately ellipsoid to triangular to bean‐shaped even within a species (e.g., fig. 6 in Aire et al., 2017; fig. 1 in Mendonca et al., 2018; fig. 17 in Vernon & Woolley, 1999), while here we have assumed an ellipsoid shape throughout. Further, we did not distinguish the mitochondrial helix from other midpiece helical structures, leading to over‐estimating mitochondrial volume when other structures were present, as the fibrous keel in the family Turdidae. These additional structures likely add noise to our analysis, but should not cause a spurious positive correlation between midpiece length and mitochondrial volume. In addition to these complexities for estimating mitochondrial volume, we note that the density of mitochondrial cristae (Mendonca et al., 2018) and the thickness of the inner membranes (Nicander & Hellström, 1967) both may also vary and impact ATP producing capacity, further impacting the use of midpiece length as an estimator of ATP production.

Our data add a layer of complexity to the results of Støstad et al. (2018), who found that species with longer spermatozoa have wider nuclei but similar nuclear volume, compared to species with shorter spermatozoa. Longer cells have wider flagella at the neck region, and the width of the flagellum at the neck correlates with the width of the nucleus immediately before the flagellum. While it may be intuitively appealing to suggest that a wider head tapering to a slightly less‐wide flagellum reduces drag through stream‐lining, this hypothesis is not likely to be valid because stream‐lining is not relevant at the spatial scale where spermatozoa operate (Humphries et al., 2008). While the cause of correlation is unclear, the evolutionary connections among nuclear and flagellar dimensions are strong. We suggest that the constriction at the opening of the females' sperm storage tubules (Mendonca et al., 2019) may be one selective force impacting sperm diameters.

Sperm swimming performance in birds has primarily been measured on glass microscope slides in fluids with relatively low viscosity. In the female reproductive tract, spermatozoa may interact closely with the walls of the female reproductive tract, rather than swimming through a purely fluid environment (mammals: Suarez, 2016; note that the vagina in birds also shows deep folds, such that spermatozoa may be expected to interact with the walls, Briskie & Birkhead, 1993). Moreover, the fluid environment is likely substantially more viscous than a standard buffer used in sperm performance assays, and viscosity is known to affect bird sperm performance in vitro (Schmoll et al., 2020), as well as having dramatic effects in some other taxa (e.g., Muto & Kubota, 2013). Thus in vitro recordings of sperm swimming performance represent a highly simplified model. In passerines, the single study to thoroughly characterize how cells move shows that they spin around their central axis much like a drill in a low viscosity environment (Vernon & Woolley, 1999). It is tempting to speculate that the helical structures of the head, as well as the mitochondrial helix, may play an important role in pushing the cell forward, much as the spiral of a screw pulls the screw into its substrate. In this case, we can hypothesize that the pitch of the gyre is important for determining forward progress. The additional structures in the midpiece, and particularly the fibrous helix that appears as a keel in Turdidae, may have implications for how effectively the sperm cell can translate spinning into forward motion, particularly in vivo. Variation among families in the fine ultrastructure of the midpiece may therefore help explain the divergent correlations between midpiece and sperm competition among bird taxonomic families (Immler & Birkhead, 2007; though note that we did not sample either of those families densely enough to draw conclusions). Our data suggest that one complete turn of a long cell would translate into greater forward displacement (assuming no slippage), since these cells have longer gyre intervals. One interspecific study supports the idea that longer cells swim faster in passerines (Lüpold et al., 2009) while another interspecific study does not (Kleven et al., 2009). For two species where we measured a substantial number of cells, flagellum length did not relate to gyre interval at the intraspecific level (supplementary online material), and indeed the correlation between cell length and swimming speed is also mixed in intraspecific studies (Cramer et al., 2015; Cramer, Garcia‐del‐rey, et al., 2021; Helfenstein et al., 2010; Immler et al., 2010; Laskemoen et al., 2010; Rojas Mora et al., 2018). Midpiece and flagellum structure may also affect swimming performance via energetics, for example, affecting ATP production and transport needs.

## 5 CONCLUSIONS

While the selective mechanism is unknown, it appears abundantly clear that sperm nucleus width, flagellum width, and flagellum length evolve in a correlated fashion. Midpiece length, while constrained to be shorter than flagellum length, may be somewhat more independent of flagellum width, though this remains to be tested. We quantitatively confirm that the flagellum tapers in passerine birds, as observed previously; we moreover show that the mitochondrial helix also tapers. Flagellum diameter is greater at the neck for species with longer flagella, consistent with longer flagella needing to be structurally stronger. Estimated mitochondrial volume varied substantially among species and was predicted by midpiece length, though we note that caution should be applied in studies where the variation in midpiece length is less (i.e., intraspecific studies or studies on taxa with relatively little midpiece variation).

## CONFLICT OF INTEREST

The authors declare no conflict of interest.

## Supporting information

Supporting information.

Supporting information.

## Data Availability

The data and code that support the findings of this manuscript, as well as the stitched images, are available at Data Dryad with DOI 10.5061/dryad.n5tb2rbzt.
