# Supplementary material for: Flagellum tapering and midpiece volume in songbird spermatozoa
Source: J Morphol. 2022 Oct 28;283(12):1577–89. doi: 10.1002/jmor.21524 (PMC9828668; doi:10.1002/jmor.21524)
Supplement: Supplementary file 2 — Supporting information. [file JMOR-283-1577-s002.docx]

***Supplementary Materials for “Flagellum tapering and midpiece volume in songbird spermatozoa”***

***Emily R. A. Cramer*, Gaute Grønstøl, Jan T. Lifjeld***

***Effect of storage time on cell length measurements***

As described in the main text, our dataset combines samples prepared for scanning electron microscopy in 2016-2017 with samples prepared in 2022; all images in the main dataset were taken in 2021-2022. To evaluate the possibility that storage of prepared SEM samples strongly impacts measurements, we compared length measurements taken from micrographs of whole cells imaged in 2016-2017 with micrographs of whole cells from the same SEM sample imaged in 2021. Whole-cell images from 2016-2017 (“early images”) were also analyzed for flagellum and mitochondrial helix width (see Additional Datasets section below), while whole-cell images from 2021 (“recent images”) were intended only as a guide to check the success of image stitching. In both cases, magnification levels differed among species; the highest magnification that allowed capture of the whole cell in a single image was used. For 26 males, we measured midpiece and tail length for at least one cell in the early and recent images; where there was more than one cell measured (primarily for the early images), the average was calculated. We then performed a paired t-test comparing the lengths. Neither midpiece length (t_25_ = -1.44, p = 0.26, mean difference = -1.53 µm, CI -4.27, 1.22 µm) nor tail length (t_25_ = 1.74, p = 0.09, mean difference = 1.92 µm, CI -0.36, 4.19 µm) differed depending on how long the SEM sample had been stored before imaging.

***Additional datasets***

To supplement the main dataset, where each species was represented by a single cell, we also measured a series of images taken during 2016-2017 (images analyzed in 2021), where there was increased replication. Since these images were taken for a different analysis (Støstad *et al.*, 2018), the number of cells per male and the number of males per species were not balanced. Taxonomic coverage was also reduced compared to the main dataset. Here, we had two types of images, generating two separate datasets.

*Single-Image cells, Methods:* Overview images of entire cells had been made in 2016-2017. Magnification varied depending on cell size between 500 and 3500 x, such that image resolution was relatively poor for long cells. These cells were measured as in the main dataset, except that image quality was too poor to directly assess the width of the major axis of the mitochondrial helix. Images were named such that neither species nor male identity was visible during measurement. Furthermore, the order of images was shuffled such that measurements of different cells for the same male were not likely to be taken consecutively. The Single Images dataset consisted of 6000 pairs of measurements (flagellum and flagellum + minor axis) distributed across 173 cells, 101 males and 36 species.

To evaluate measurement repeatability, we fully re-measured 55 entire cells from 12 males of two species (chosen because they had a large number of cells available), on different days. Here we used rptR and controlled for measurement type (flagellum or flagellum + minor axis), species, and male identity as fixed effects. Repeatability was 0.739 (SE 0.008, CI 0.721 – 0.753, p< 0.001). The average difference between measurements was 5.8 ± 5.1% for the flagellum and 15.4 ± 14.7% for the minor axis.

To investigate how measures changed across the length of the flagellum, and as a function of the total flagellum length, we ran models as described in the main text, where each measurement along the cell was included as an observation. Response variables (in separate models) were flagellum width, minor axis width, or the interval between successive gyres. Predictors were flagellum length for that individual cell (to estimate the relationship between flagellum length and the width measures; centered and scaled by dividing by 100) and longitudinal position (to assess tapering). We included cell ID, male ID, and species as three separate random effects. Note that in some modelling frameworks these would best be considered nested random effects, but with MCMCglmm nesting creates a variance component for each observed level of the nested effects (e.g., each cell), which is not desirable. (Parameter estimates using a properly nested model and accounting for phylogeny in the Bayesian package brms were highly similar, but those models had problems with divergent results and therefore are not shown).

For the gyre interval analysis only, we examined whether the same patterns were evident at an intraspecific level for the two species where we had substantial intraspecific sample size (the willow warbler *Phylloscopus trochilus* and the common redstart *Phoenicurus phoenicurus*). We did not attempt this for the width measurements, because of relatively high measurement error for widths. For each of these species separately, we created a linear mixed model with lme4 (Bates *et al.*, 2015), with statistical significance assessed with lmerTest (Kuznetsova *et al.*, 2017). The gyre interval was the response variable, and models included a random effect of cell identity nested within male identity. Fixed effects were longitudinal position and the flagellum length of the individual cell, as well as an interaction between the two (removed if it was not significant, i.e., p > 0.05).

*Single-Image cells, Results:* As in the main dataset, flagellum diameter decreased along the length of the flagellum and (Table S3, Figure S1), and there was a significant interaction between flagellum length and position on the flagellum, such that there was a tendency for cells with longer flagella to be wider at the neck end (position 0 in Figure S1) but no trend between flagellum length diameter at the tail end. For the minor axis of the mitochondrial helix, diameter decreased with longitudinal position along the flagellum, but unlike in the main dataset, minor axis diameter was smaller in cells with longer flagella (Table S3). Note, however, that minor axis had relatively high measurement error in the Single Images dataset. The interval between gyres on average was longer in species with longer midpieces, implying fewer wraps of the midpieces per length of midpiece (Table S3).

*
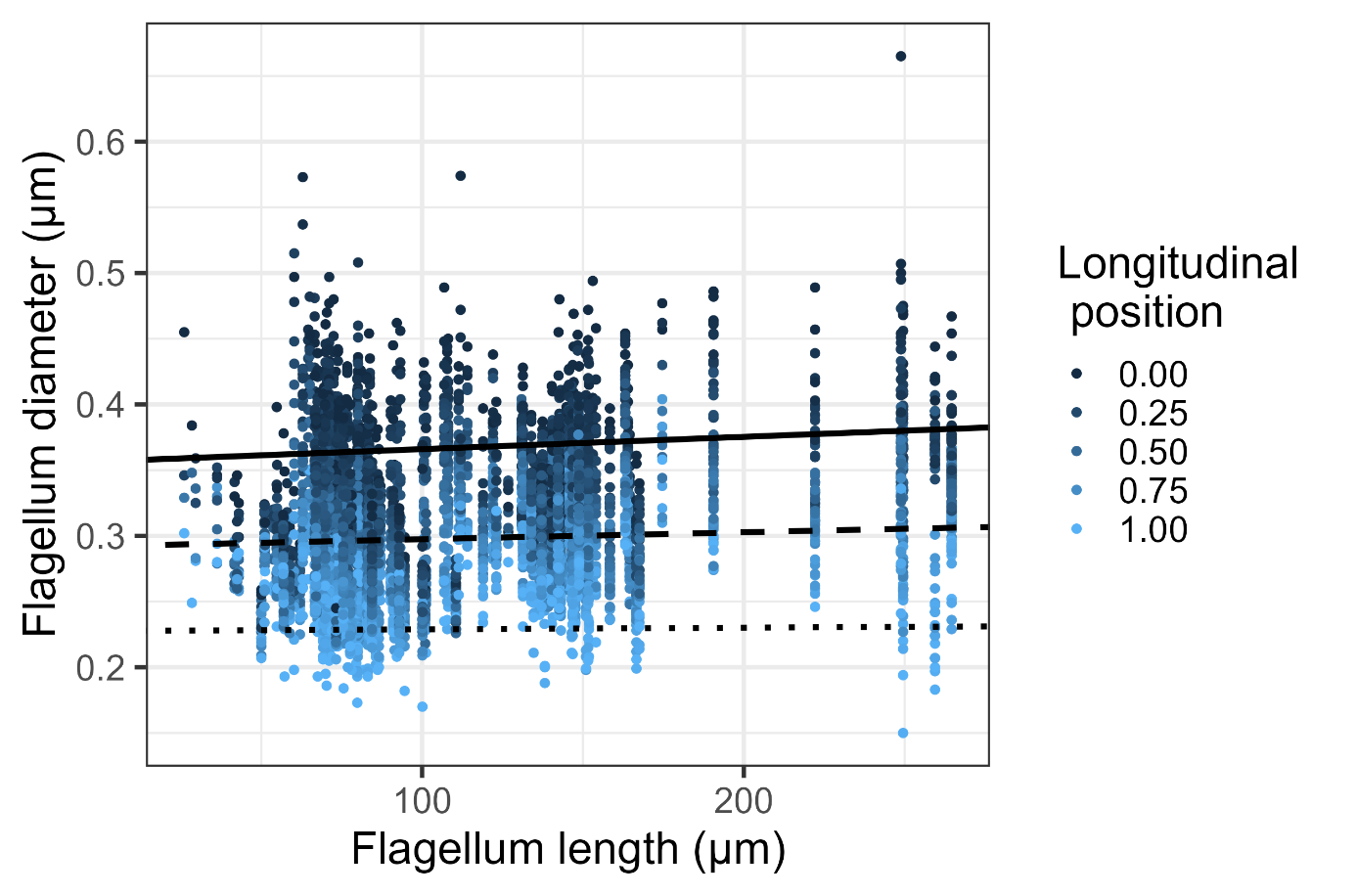
*

*Figure S1. Flagellum diameter change over longitudinal position (color gradient, 0 = neck, 1 = tail), and with total flagellum length, from the single-images dataset. The lines are estimates from the statistical analysis controlling for phylogeny, where longitudinal position is 0 (solid; neck), 0.5 (dashed) or 1 (dotted; tail). Each cell is represented by multiple points (on point per measurement location).*

| Table S3. Statistical results relating flagellum diameter, minor axis diameter of the mitochondrial helix, and gyre interval to longitudinal position along the cell, and flagellum length. Estimated values are means from the posterior distribution of the Bayesian model. Flagellum length was centered and scaled by dividing by 100. Longitudinal position was between 0 and 1 for all cells. Here df indicates effective sample size after the Markov chain process in MCMCglmm, controlling for shared phylogenetic history. These results refer to the Single Images dataset, for comparison to Table 1 in the main text. | | | |
| --- | --- | --- | --- |
| Response | Predictor | Estimate [CI], statistics | Lambda (mean [CI]) |
| Flagellum diameter | (Intercept) | 0.36 [0.33, 0.39], df = 368, p = 0.003 | 0.73 [0.61 – 0.84] |
|  | Longitudinal position | -0.13 [-0.13, -0.13], df = 337, p = 0.003 |  |
|  | Flagellum length | 0.01 [-0.01, 0.03], df = 394, p = 0.31 |  |
|  | Flagellum length * Longitudinal position | -0.01 [-0.01, -0.01], df = 247, p = 0.003 |  |
| Minor axis of mitochondrial helix † | (Intercept) | -1.75 [-2.13, -1.39], df = 631, p = 0.003 | 0.72 [0.61 – 0.82] |
|  | Longitudinal position | -0.70 [-0.73, -0.69], df = 394, p = 0.003 |  |
|  | Flagellum length | -0.38 [-0.47, -0.28], df = 394, p = 0.003 |  |
| Gyre interval† | (Intercept) | 1.42 [1.27, 1.56], df = 568, p = 0.003 | 0.75 [0.64 – 0.84] |
|  | Flagellum length | 0.18 [0.10, 0.27], df = 394, p = 0.01 |  |
|  | Longitudinal position | -0.12 [-0.12, -0.11], df = 394, p = 0.003 |  |
|  | Flagellum length * Longitudinal position | 0.12 [0.11, 0.13], df = 394, p = 0.003 |  |

† indicates that the response variable was log-transformed to improve normality of residuals

Patterns with gyre interval were less clear-cut at the intraspecific level. For willow warblers, the gyre interval became shorter toward the tail end of the cell (main effect ± SE, -2.12 ± 0.30, t_2687_ = -6.95, p < 0.001), although this depended on the flagellum length of the cell (interaction 0.02 ± 0.00, t_2686_ = 4.52, p < 0.001; no scaling or transformation of variables). The main effect of flagellum length was not significant (0.004 ± 0.006, t_28_ = 0.62, p = 0.54; n = 48 cells and 9 males). In common redstarts, gyre interval also became shorter towards the tail (-0.05 ± 0.01, t_3879_ = -8.77, p < 0.001), but flagellum length had no effect (0.002 ± 0.003, t_37_ = 0.76, p = 0.45; n = 39 cells, 6 males; interaction term removed from model).

*Background cells, Methods:* In addition to the Single Images dataset, we also had access to higher-resolution images, of cell portions that appeared in the background of sperm head images used by Støstad et al. (2018). Here magnification and image resolution was high (between 3500 and 12,000 x, mostly between 9000 and 11,000 x), but positional information was lacking. We assume that each background cell measured was a different cell, although this could not be verified across different images. The Background Cells dataset consisted of 461 complete sets of measurements (flagellum, flagellum + minor axis, and major axis) from 171 males and 39 species.

To evaluate repeatability, we marked and re-measured (4 measures total, on separate days) major axis, flagellum, and flagellum + minor axis from each of 11 cells (in 8 images, all from the same male). We then calculated repeatability as in the main dataset. Note that repeatability here represents the percent of variation that is between-cell, compared to total variation. Repeatability was 0.517 (SE = 0.128, 95% CI 0.212 - 0.701, p > 0.001), and we suggest that this low value represents low variation among cells rather than high measurement error. The difference between the largest and smallest values for repeated measurements of the same point was only 6.0% ± 4.2% (mean ± SD of the absolute value of the difference).

Species names were visible during the measuring of the Background Cells dataset. As for the few labelled cells in the main dataset, we contend that this was unproblematic, as there were no strong expectations linked to species identity.

For the Background Cells dataset, positional information was missing. We therefore used model results from the main dataset to generate predictions about how flagellum length should relate to major axis, minor axis, and flagellum width within the Background Cells dataset, where we assume that the measurements are taken at random points along the cell. We use the mean flagellum measurement from that individual male (measured via light microscopy on different spermatozoa) as the flagellum length measurement. For 8 males, light microscopy data were unavailable, and here we instead used the species mean value. Two measurements (out of 461) resulted in slightly negative calculations for the minor axis diameter. These were removed prior to analysis. One case appeared to result from minor variation in the thickness of the flagellum, causing the flagellum-only measurement to be greater than the flagellum + minor axis measure taken for the same gyre. The other case likely resulted from measurement error, when a flagellum-only measurement likely included some mitochondrial helix (which at this point on the cell was still wrapping around the flagellum to a considerable degree).

*Background cells results:* Results from the background cells matched predictions from the main results, when assuming background cells were randomly positioned along the flagellum (Table S4). Specifically, only flagellum diameter, but neither diameter of the mitochondrial helix, was significantly related to mean flagellum length for the male (or species).

| Table S4. Statistical results relating flagellum diameter or the major or minor axis diameters of the mitochondrial helix to flagellum length in the background cells dataset. Flagellum length (from light microscopy on the same male or a conspecific) was centered and scaled by dividing by 100. Here df indicates effective sample size after the Markov chain process in MCMCglmm, controlling for shared phylogenetic history. Compare to Table 1 in the main text. | | | |
| --- | --- | --- | --- |
| Response | Predictor | Estimate [CI], statistics | Lambda (mean [CI]) |
| Flagellum diameter | (Intercept) | 0.28 [0.26, 0.32], df = 394, p = 0.003 | 0.43 [0.26 – 0.62] |
|  | Flagellum length | 0.03 [0.02, 0.05], df = 394, p = 0.003 |  |
| Minor axis of mitochondrial helix | (Intercept) | 0.15 [0.09, 0.21], df = 394, p = 0.003 | 0.86 [0.77 – 0.94] |
|  | Flagellum length | 0.00 [-0.03, 0.03], df = 282, p = 0.88 |  |
| Major axis of mitochondrial helix | (Intercept) | 0.21 [0.18, 0.24], df = 394, p = 0.003 | 0.43 [0.22 – 0.63] |
|  | Flagellum length | -0.00 [-0.02, 0.01], df = 394, p = 0.72 |  |

***Mapping midpiece structure qualitatively from the main dataset***

As described in the introduction, the midpiece of several species is known to have a granular helix and/or a fibrous helix in addition to the mitochondrial helix. Further, the mitochondrial helix is described as forming a ring around the flagellum for some species but not others. Using a fully blinded dataset of the stitched 4000 X images (i.e., the main data images), two observers independently categorized the cells. The neck region was assessed for whether 1) the mitochondrial helix appeared to widen into a ring encompassing the full flagellum circumference; 2) the first approximately 1-2 gyres of the helix had a substantially different texture (presumed to be granular helix replacing the mitochondrial helix) and/or showed an abrupt transition point with the mitochondrial helix (Figure 6A); 3) there was an additional, symmetrical structure surrounding the mitochondrial helix (presumed to be a granular helix; see Figure 6B); or 4) none of these occurred. Separately, the apparent cross-sectional shape of the mitochondrial helix was assessed at the neck, in the remainder of the proximal half of the cell, and in the distal half of the cell. For each of these three portions, the helix was categorized as 1) approximately ellipsoid (including a relatively flat ellipse; Figure 6B and Figure 2); 2) appearing divoted or bipartite, (figure 6C; or 3) with a sharp keel (Figure 6D). The two observers then discussed and reconciled scores that disagreed.

SEM imaging is a less robust method than, for example, transmission electron microscopy (TEM), for assessing whether additional structures are present in the midpiece. Because TEM was outside the scope of this study, the following descriptions should be hypotheses warranting further investigation, rather than as a proper description for each species. Moreover, more cells should be examined per species for a formal description.

Many species had a putative granular helix that either replaced or encompassed the mitochondrial helix at the neck (Table S1), and these species appeared fairly scattered among taxonomic families. Our independent categorization (blind with respect to species) matched the results of Tripepi and Perrotta (1991), who used TEM and examined sperm structure throughout spermatogenesis in seven of the same species studied here (see Table S1).

The apparent cross-sectional shape of the mitochondrial helix appeared more consistent within taxonomic families. Several taxonomic families showed no evidence of additional structures accompanying the mitochondrial helix (for example, Paridae, Troglodytidae, Passeridae, and Fringillidae; Table S1). Many species’ mitochondrial helices had either a triangular or a divoted shape at the neck and or/central portion, which often became more ellipsoid toward the tail (Table S1). In particular, we note that all *Turdus* species, as well as one species each from *Acrocephalus* and *Regulus* have an approximately triangular cross-sectional shape; in *Turdus* this is likely due to a peripheral fibrous helix, but whether there is a similar structure causing the triangular aspect in the other genera requires further work. In addition, many other species had a divoted appearance, at least at the neck end of the mitochondrial helix, which could represent an additional structure located between the mitochondrial helix and the flagellum as in Southern anteater-chat and European starling (Vernon & Woolley, 1999; Jamieson *et al.*, 2006), or perhaps the microtubular helix present during spermatogenesis left an impression on the cell after the microtubular helix itself sloughed off (Bawa *et al.*, 1990). We recommend further TEM work to elucidate whether these shape differences are caused by additional structures or simply variation in the mitochondrial helix itself. In addition, examining intra-ejaculate and inter-male variation would be of interest.
